# *Mucuna pruriens,* a Possible Treatment for Depressive Disorders

**DOI:** 10.3390/neurolint16060112

**Published:** 2024-11-16

**Authors:** Alfonso Mata-Bermudez, Araceli Diaz-Ruiz, Luis Ricardo Silva-García, Eduardo Manuel Gines-Francisco, Roxana Noriega-Navarro, Camilo Rios, Héctor Alonso Romero-Sánchez, Diego Arroyo, Abraham Landa, Luz Navarro

**Affiliations:** 1 Departamento de Fisiología, Facultad de Medicina, Universidad Nacional Autónoma de México, Ciudad de México 04510, Mexico; alfonsomata24@hotmail.com (A.M.-B.); roxnn77@gmail.com (R.N.-N.); qfbdiegoarroyo@gmail.com (D.A.); 2 Departamento de Atención a la Salud, Universidad Autónoma Metropolitana Unidad Xochimilco, Ciudad de México 04960, Mexico; rich.sil.garcia@gmail.com (L.R.S.-G.); edy16263@gmail.com (E.M.G.-F.); alonsoromero1278@gmail.com (H.A.R.-S.); 3 Departamento de Neuroquímica, Instituto Nacional de Neurología y Neurocirugía Manuel Velasco Suarez, Ciudad de México 14269, Mexico; adiaz@innn.edu.mx; 4 Laboratorio de Neurofarmacología Molecular, Departamento de Sistemas Biológicos, Universidad Autónoma Metropolitana Unidad Xochimilco, Ciudad de México 04960, Mexico; camrios@yahoo.com.mx; 5 Dirección de Investigación, Instituto Nacional de Rehabilitación Luis Guillermo Ibarra, Calzada Mexico-Xochimilco 289, Arenal de Guadalupe, Ciudad de México 14389, Mexico; 6 Departamento de Microbiología y Parasitología, Facultad de Medicina, Universidad Nacional Autónoma de México, Ciudad de México 04510, Mexico; landap@unam.mx

**Keywords:** *Mucuna pruriens*, depression, catecholamines, oxidative stress, nitric oxide, cortisol, inflammatory response

## Abstract

Depression is a mental disorder that depicts a wide variety of symptoms, including mood and cognitive alterations, as well as recurrent thoughts of death or suicide. It could become the second leading cause of premature death or disability worldwide. Treatments with conventional antidepressants have several limitations in terms of effectiveness, side effects, and high costs. Therefore, medicinal plants such as *Mucuna pruriens* are potent candidates for treating depressive disorders. This review shows a compendium of evidence supporting the antidepressant effect of the *Mucuna pruriens* plant in diverse animal models. This includes the mechanisms of action underlying the antidepressant activity of the treatment concerning dopamine, serotonin, norepinephrine, reactive oxygen species, nitric oxide, cortisol, and inflammation. Clinical trials are needed to study the efficacy and safety of *Mucuna pruriens* for depression.

## 1. Introduction

Depression is a mood disorder that generates a persistent feeling of sadness, constant negative thoughts, apathy, lack of energy, cognitive distortions, nihilism, and an inability to enjoy everyday life events [1]. The DSM V (Diagnostic and Statistical Manual of Mental Disorders) classifies depressive disorders according to their duration, timing, and possible etiology [2]. The spectrum of depressive disorders includes depression in bipolar I disorder, depression in bipolar II disorder, mixed depression, agitated depression, atypical depression, melancholic depression, brief recurrent depression, minor depressive disorder, seasonal depression, and dysthymic disorder [3]. Symptoms of depressive disorders include low mood, anhedonia, decreased body weight, sleep disturbances, motor agitation, fatigue, feelings of worthlessness or guilt, decreased ability to concentrate, and suicidal ideation. Such symptoms can cause distress and impairment in social and occupational functionality [1]. In 2019, depressive disorders were among the most common mental health issues globally, affecting an estimated 279.6 million people. The COVID-19 pandemic exacerbated this burden, leading to a 27.6% increase in global cases due to social isolation, reduced mobility, and heightened stress. This pandemic-driven rise disproportionately affected women and younger populations, underscoring the need for enhanced mental health interventions and preventive strategies to manage depression’s long-term impact [4,5].

Current evidence suggests that conventional antidepressants, particularly second-generation ones, have proven efficacy in the treatment of major depressive disorder (MDD), especially in moderate to severe episodes. However, many patients do not respond adequately to first-line treatment, requiring therapeutic adjustments such as switching or combining medications and using adjuvant treatments to improve clinical outcomes [6]. The commonly used pharmacological treatments are based on three different mechanisms of action: (1) blockade of presynaptic monoamine transporter proteins, whose function is to remove the released neurotransmitter from the extracellular space; (2) inhibition of monoamine oxidase, responsible for the degradation of monoamines; (3) inhibition or excitation of pre- or postsynaptic receptors, which regulate the release of monoamine neurotransmitters [7]. However, it has been reported that around 20% of patients with depression do not respond to these treatments [8]. Antidepressant drugs often cause numerous adverse reactions and are generally expensive [9]. Consequently, it is common for patients not to adhere to the treatment, thus reducing therapeutic success.

In response to the current problem of the lack of effective treatment for depressive disorders, proposals for new therapeutic alternatives that are effective, safe, and affordable for the management of this condition have arisen. In this same sense, interest has grown in studying medicinal plants with antidepressant activity [9]. Such is the case of *Mucuna pruriens* (*M. pruriens*), commonly known as the velvet bean, belonging to the *Fabaceae* family [10]. The extension of *M. pruriens* cultivation generally includes tropical and subtropical regions worldwide since it is considered an important source of protein (23–35%), has good digestibility, and has low production costs [11]. The safety and efficacy of *M. pruriens* have been confirmed in various studies. For example, 50 µg/mL of the extract did not show toxic effects on human cervical adenocarcinoma (HeLa) cells [12]. An animal study demonstrated that the oral administration of 100 mg/kg of flavonoids isolated from *M. pruriens* had no toxic effect and did not cause the death of animals throughout the trial [13]. Intraperitoneal administration of a mixture of plant extracts containing *M. pruriens* (25 mg), *Tribulus terrestrial* (300 mg), *Whitania somnifera* (100 mg), and *Argvreia speciosa* (60 mg) for 6 to 8 weeks in mice did not exhibit apparent toxic effects; the authors revealed that the formulation containing the extract of *M. pruriens* is safe up to a dose of 5000 mg/kg [14].

In contrast, there are only two records of an outbreak of acute toxic psychosis attributed to the legume *M. pruriens* in November 1989 in the Memba district, Nampula province, Mozambique. The authorities reported the existence of 203 cases of psychosis during six weeks, affecting mainly women of reproductive age. It should be noted that all affected patients recovered within two weeks after the administration of intravenous chlorpromazine [15]. In a case study published on Réunion Island, a French tropical island nation in the Indian Ocean close to Mauritius, a 58-year-old woman experienced nausea, vomiting, and diarrhea after consuming about five raw *M. pruriens* seeds. She also experienced confusion, hallucinations, and amnesia. Her vital signs were within normal limits; the only lingering condition was nausea. This example demonstrates how eating raw or undercooked *M. pruriens* seeds can result in neurological abnormalities and severe gastrointestinal symptoms [16].

Currently, the therapeutic dose in humans has yet to be standardized. However, there is growing evidence supporting the safety and efficacy of using *M. pruriens* extracts in patients with Parkinson’s disease (PD) [17,18] and infertility [19]. In addition, the extract has been widely used in traditional medicine of diverse cultures worldwide, demonstrating anti-inflammatory, antiepileptic, antimicrobial, and antioxidant properties [20].

Several research groups have proposed using *M. pruriens* for the treatment of depressive disorders based on the feasibility of its modulating action on the systems that participate in generating depressive behavior [21]. This review will focus on presenting and discussing the potential antidepressant properties of the *M. pruriens* plant and the possible mechanisms of action involved in the side effects.

## 2. *Mucuna pruriens*

The *M. pruriens* plant is a climbing legume native to southern China and eastern India. It has traditionally been used as a food source by certain ethnic groups in various countries [11]. It has also been a nutraceutical and pharmaceutical product [10] for treating different diseases and conditions such as malaria, cancer, epilepsy, PD, diarrhea, helminthiasis, ulcers, infertility, elephantiasis, snake bites, and scorpion stings [12]. The high nutritional value of the plant is attributed to the presence of numerous amino acids such as threonine, proline, tyrosine, phenylalanine, tryptophan, serine, lysine, histidine, and arginine [10]. In addition, it has a significant amount of dietary fiber, fatty acids, carbohydrates, vitamins, and minerals [22].

**Figure 1:**
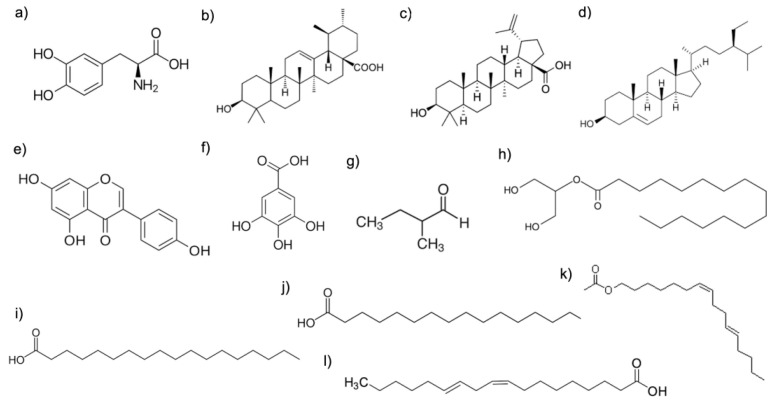
Chemical structures of the main bioactive compounds found in the *M. pruriens* plant. (**a**) L-3,4-dihydroxyphenylalanine (L-Dopa); (**b**) ursolic acid; (**c**) betulinic acid; (**d**) genistein; (**e**) octadecanoic acid; (**f**) gallic acid; (**g**) 2-methylbutanal; (**h**) 2-palmitoylglycerol acid 2-hydroxy-1-(palmitoylglycerol); (**i**) beta-sitosterol; (**j**) n-hexadecanoic acid (palmitic acid); (**k**) (Z,E)-7,11-hexadecadien-1-yl acetate; (**l**) 9,12-octadecadienoic acid.

Among the main bioactive compounds found in the plant, L-3,4-dihydroxyphenylalanine (L-Dopa), ursolic acid, and betulinic acid are the most remarkable; they have been shown to have significant biological activity [20]. Other compounds found in the plant include 2-methyl butanal, *n*-hexadecanoic acid (palmitic acid), 9,12-octadecadienoic acid, octadecanoic acid, hexadecanoic acid 2-hydroxy-1-(palmitoylglycerol), and (Z, E)-7,11-hexadecadien-1-yl acetate [23] (Figure 1). The use of specific bioactive compounds with antidepressant activity has been documented in diverse animal models (Table 1).

Numerous experimental tests have identified several effects on the nervous system attributable to the *M. pruriens* plant due to its L-Dopa content.

**Figure 2:**
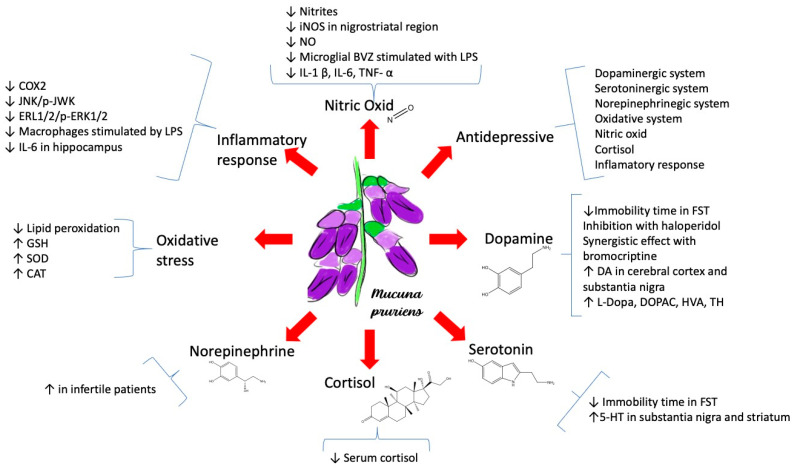
Mechanisms involved in the pharmacological properties of *M. pruriens*. COX-2, cyclooxygenase-2; ERK, extracellular-signal-regulated kinase; LPS, lipopolysaccharide; IL-6, interleukin-6; GSH-Px, glutathione peroxidase; SOD, superoxide dismutase; CAT, catalase; iNOS, inducible nitric oxide synthase; NO, nitric oxide; IL-1β, interleukin-1β; TNF-α, tumor necrosis factor; FST, forced swim test; DA, dopamine; DOPAC, dihydroxyphenylacetic acid; HVA, homovanillic acid; TH, tyrosine hydroxylase.

Among the pharmacological properties of the plant are: (1) anti-inflammatory capacity [24]; (2) modulation of the dopaminergic system [21]; (3) modulation of the serotonergic and noradrenergic systems [25]; (4) antioxidant effect [26]; and (5) neuroendocrine modulation [27]. These pharmacological properties are closely related to the antidepressant effect of the *M. pruriens* plant, which is described in more detail below and in Figure 2.

**Table 1:** Effect of bioactive compounds of the *M. pruriens* plant on behavior associated with depression in animal models. 5-HT: serotonin; PCPA: p-chlorophenylalanine; SCH23390: (R)-(+)-7-chloro-8-hydroxy-3-methyl-1-phenyl-2,3,4,5-tetra-hydro-1H-3-benzazepine hydrochloride; FST: forced swim test; TST: tail suspension test; ICR: Institute for Cancer Research; p.o.: oral administration; i.p.: intraperitoneal administration.

Compound	Species	Inducing Agent/Model	Dose	Effect	Reference
Genistein	Wistar Rats	Varying schedule of minor stressors/ Mild chronic stress	100 mg/kg, p.o. for 45 days	↑ sucrose preference ↑ monoamines and ↓ cortisol serum levels ↓ Immobility time in FST	Chang et al., 2021 [28]
Genistein	ICR Mice	FST and TST/Depression	5–45 mg/kg, p.o. for 21 days	↓ Immobility time in FST and TST ↑ monoamines level ↓ monoamine oxidase activity in the brain	Hu et al., 2017 [29]
		Chemical depletion of 5-HT with PCPA (300 mg/kg/day), serotonin synthesis inhibitor		Reverted ↓ immobility time in FST and TST	
Ursolic acid	Swiss mice	Depression by TST and FST	0.01 and 0.1 mg/kg, and 10 mg/kg p.o., respectively	↓ Immobility time in FST and TST	Machado et al., 2012 [30]
		Administration of SCH23390 (0.05 mg/kg, s.c. dopamine D1 receptor antagonist) and sulpiride (50 mg/kg, i.p. dopamine D2 receptor antagonist)	0.1 mg/kg, p.o.	Reverted ↓ immobility time in TST	
Ursolic acid	Swiss mice	Multiple stressors randomly/ Unpredictable chronic stress	0.1 mg/kg, p.o. once a day for 7 days	↓ Immobility time in FST and TST	Colla et al., 2021 [31]
Gallic acid	Sprague Dawley rats	Repeated administration of sodium arsenite (2.5 mg/kg, i.p.)/Depression	50 and 100 mg/kg, i.p. once a day for 4 weeks	↓ Immobility time in FST	Samad et al., 2019 [32]
Gallic acid	Balb/c mice	Bilateral common carotid artery occlusion/ischemic stroke/secondary form of depression	25 and 50 mg/kg, i.p. once a day for 7 days	↓ Immobility time in FST and TST ↑ Sucrose preference	Nabavi et al., 2016 [33]
beta-sitosterol	ICR mice	FST and TST/Depression	2.5 mg/kg, i.p. single dose	↓ Immobility time in FST and TST	Yin et al., 2018 [34]

## 3. Antidepressant Effect Induced by *Mucuna pruriens* in Animal Models

The antidepressant effect induced by the *M. pruriens* plant in different medical conditions has been documented in various animal models (Table 2). These include the tail suspension test, the forced swim test, and the sucrose or saccharin consumption preference test [21,35]. The tests offer a broad picture of animal behavior and associate such behavior with the onset, maintenance, and regulation of depressive disorders in humans [36]. Findings in experimental models have shown that acute and chronic treatment with *M. pruriens* has significant antidepressant activity. It decreases the immobility time in the forced swim and tail suspension tests and significantly increases the preference for sucrose consumption [21,24,25,37]. These reports suggest that *M. pruriens* influences depressive behavior in a similar way to imipramine, a drug used to treat depression [21]. Also, they demonstrated that both acute and chronic treatments have a significant effect on depressive behavior in the animals evaluated.

## 4. *Mucuna pruriens* and Dopamine

Various studies have shown that physiological alterations involved in the generation and development of depressive behavior are closely related to an alteration of the dopaminergic system, especially with the reduction in dopamine (DA) signaling in diverse brain regions [7], which constitutes the mesocortical pathway and the mesolimbic pathway [38,39,40]. Although the mechanism for generating depressive behavior is still not entirely clear, there is evidence that treatment with *M. pruriens* (100 mg/kg and 200 mg/kg, p.o. for seven days) in Swiss mice reversed behavior associated with depression. It significantly reduced the immobility time in the forced swim test and the tail suspension test until reaching values close to those obtained by the positive control (Imipramine, 10 mg/kg, p.o.). This action was inhibited by administering haloperidol (dopamine D2 receptor antagonist, 0.1 mg/kg, i.p.) 30 min after the last dose of *M. pruriens* [21].

**Table 2:** Effects of treatment with *M. pruriens* on behavior associated with depression in animal models. FST: forced swim test; TST: tail suspension test; p.o.: oral administration; i.p.: intraperitoneal administration.

Species	Inducing Agent/Model	Treatment with *Mucuna pruriens*	Effect	Reference
Wistar rats	Cafeteria diet/Obesity	750 mg/kg, p.o. for 8 weeks	↓ Immobility time in FST ↑ Sucrose consumption	Tavares et al., 2020 [24]
Swiss mice	FST, TST, and chronic unpredictable mild stress/Depression	100 mg/kg and 200 mg/kg, p.o. for 7 days	↓ Immobility time in FST and TST ↑ Sucrose consumption	Rana and Galani 2014 [21]
Swiss mice	FST and TST/ Depression	200 mg/kg, p.o. for 7 and 14 days	↓ Immobility time in FST and TST	Patel and Galani 2013 [25]
Swiss mice	FST and TST/ Depression	10–20 mg/kg, i.p. single dose 45 min before the observation period	↓ Immobility time in FST and TST	Pati et al., 2010 [41]
Swiss mice	FST and TST/ Depression	100, 200, and 300 mg/kg, p.o. 1 h before the observation period	↓ Immobility time in FST and TST	Patil Rupali and Ahmad Azharuddin 2021 [42]
Swiss mice	FST and TST/ Depression	200 mg/kg, p.o. single dose	↓ Immobility time in FST and TST	Karekar et al., 2023 [43]
Wistar rats	Traumatic brain injury	50 mg/kg i.p. per day for five days	↓ Immobility time in FST	Mata-Bermudez et al., 2024 [37]

On the other hand, the combined administration of bromocriptine (a dopamine D2 receptor agonist, 2 mg/kg, i.p.) and *M. pruriens* (200 mg/kg, p.o.) showed significant potentiation by decreasing immobility time in the forced swim test and the tail suspension test [21]. Another study indicated that simultaneous administration of *M. pruriens* (10 mg/kg, i.p.) with the selective inhibitor of norepinephrine and dopamine reuptake, bupropion (20 mg/kg, i.p.), potentiated the antidepressant effect by significantly decreasing the time of immobility in the forced swim test and the tail suspension test [41]. These data suggest that *M. pruriens* could modulate the dopaminergic system. Furthermore, the administration of an oral formulation of *M. pruriens* (10 g/kg) increased DA concentrations in the cerebral cortex in rats [44], while a minor dose (100 mg/kg) increased concentrations of DA, dihydroxyphenylacetic acid (DOPAC), homovanillic acid (HVA), and tyrosine hydroxylase (TH) immunoreactivity in 1-methyl-4-phenyl-1,2,3,6-tetrahydropyridine-(MPTP)-injured mice [45]. *M. pruriens* has demonstrated neuroprotective properties in other animal PD models [20].

Many of the neuroprotective effects of *M. pruriens* have been attributed to L-Dopa, a metabolic precursor of DA, found in significant amounts in the *M. pruriens* plant [46]; this property is the rationale for its wide use in treating symptoms of PD [47]. About 80–90% of L-Dopa is absorbed in the small intestine with the help of the L-amino acid transport system. However, due to the presence of the aromatic L-amino acid decarboxylase enzyme (AADC), the bioavailability is approximately 5–10% of L-Dopa, and only 1% makes it through the blood–brain barrier [47,48,49]. The maximum serum concentrations are reached between 0.5 and 2 h after oral administration. Once in the system, L-Dopa is converted to DA via AADC [50].

The metabolism of DA is carried out by oxidation, where the enzyme monoamine oxidase (MAO) participates and results in the production of DOPAC. The metabolism of L-Dopa can be carried out through transamination, where the enzyme catechol-O-methyltransferase (COMT) plays a key role in the synthesis of HVA [51].

Reports indicate that unilateral injury with 6-hydroxydopamine (6-OHDA) yields a 95% loss of DA in the striatum of rats with PD. It also increased the immobility time in the forced swim test, suggesting that the decrease in DA concentrations favors the increase in depression-like behavior [52]. Oral administration of *M. pruriens* (5 g/kg) significantly restored the endogenous L-Dopa concentration in the substantia nigra and striatum [53]. Treatment with L-Dopa (50 mg/kg s.c.) attenuates depressive behavior in the forced swim test in rats with nicotine-induced withdrawal syndrome [54].

## 5. *Mucuna pruriens* and Serotonin

There are various hypotheses about the genesis of depressive disorders. One of them, and one of the most accepted, postulates that depression is caused by an alteration in the concentration of serotonin (5-HT) in the brain [5]. This finding has been strengthened by the administration of tricyclic antidepressants and selective 5-HT reuptake inhibitors, which increase the levels of these serotonergic metabolites [55].

Evidence of the involvement of the serotonergic system in the pathophysiology of depressive disorders emerged from studies that used acute dietary manipulation. The latter produced a transient decrease in 5-HT activity in the brain through decreased availability of its precursor amino acid, tryptophan [55]. In consequence, abnormalities in 5-HT concentrations have been repeatedly reported to be strongly associated with the onset of depressive behavior [56].

Currently, the pharmacological treatment of depressive disorders is preponderantly based on the use of selective 5-HT reuptake inhibitors (SSRIs) and dual 5-HT and norepinephrine reuptake inhibitors (SNRIs). Such a therapeutic approach aims to increase the concentration of 5-HT in the brain and subsequently elicit an antidepressant response [57]. However, more than 40% of patients treated for major depressive disorder do not respond to drug therapy [58]. Findings in experimental models have exhibited that chronic treatment with the hydroalcoholic extract of *M. pruriens* seeds (200 mg/kg, p.o.) produced an antidepressant effect. It decreased the immobility time in the forced swim test and the tail suspension test in mice compared to the control; this effect was evident at 7 and 14 days after administration of the extract. The effect was reversed by p-chlorophenylalanine (p-CPA, 5-HT synthesis inhibitor), suggesting that the antidepressant action of *M. pruriens* is regulated by interaction with the serotonergic system [25]. Previous studies have reported that *M. pruriens*, in addition to containing a significant amount of L-Dopa, also contains a significant amount of 5-HT, with concentrations predominantly found in seeds [59]. Moreover, treatment with *M. pruriens* (5 g/kg) increases 5-HT levels in the striatum and substantia nigra of PD rats [53].

## 6. *Mucuna pruriens* and Norepinephrine

Norepinephrine has been related to regulating motivation, alertness, and wakefulness. All these functions have been observed as altered or diminished in patients with depressive disorders [60]. Norepinephrine is synthesized from the amino acid tyrosine, primarily in the *locus coeruleus*, located on the floor of the fourth ventricle, in the rostral pons, followed by the ventral tegmental area. According to studies carried out in patients with depression, noradrenaline deficiency can be unfolded as intrinsic due to decreased production or secondary as a result of chronic stimulation of the noradrenergic system, for example, due to chronic stress [61].

Post-mortem studies carried out on suicide victims have detected changes in the density of β-adrenergic receptors in the frontal cortex. Some tricyclic antidepressants with action on norepinephrine, such as desipramine, have shown utility by improving the reuptake of norepinephrine in the synaptic space, and MAO enzyme inhibitor antidepressants suppress norepinephrine metabolism [62].

The *M. pruriens* treatment has outlined its capacity to increase noradrenaline concentrations in infertile patients [63]. Furthermore, an animal study indicated that it could increase noradrenaline concentrations in a rat model of PD [44].

## 7. *Mucuna pruriens* and Oxidative Stress

The antioxidant properties of the *M. pruriens* plant can be attributed to the presence of a vast number of phenolic compounds (flavonoids and tannins) in its composition. These compounds stand out for their ability to capture reactive oxygen species (ROS) such as superoxide anion (O_2_^−^), hydrogen peroxide (H_2_O_2_) [26], hydroxyl (OH^−^), and nitric oxide (NO) [64], in addition to decreasing lipid peroxidation [65,66,67] in several pathologies (Table 3).

In recent years, interest in studying the relationship between oxidative stress and the development of depressive disorders has thrived. Depression is considered a disorder of multifactorial origin that may be associated with an imbalance in the production of ROS and a decrease in the antioxidant protection systems [68,69,70]. Various studies on patients with major depression indicate that there is a considerable decrease in enzymes with great antioxidant power; this includes superoxide dismutase (SOD), glutathione peroxidase (GSH-Px), and catalase (CAT) [71].

Also, high concentrations of lipid peroxidation products in the hippocampus and prefrontal cortex of animals that were subjected to experimental models of depression [72] have been observed.

Compounds with great antioxidant potential can decrease the behavior associated with depression by modulating oxidative stress in rats [73]. These findings suggest that the use of this type of compound may be effective for the treatment of depressive disorders. An in vitro study revealed that the hydroalcoholic extract of the *M. pruriens* plant decreased lipid peroxidation; the latter was initiated by the increased production of O_2_^−^ radicals and OH radicals in rat liver [67]. Another study reported that treatment for six months with the hydroalcoholic extract of the *M. pruriens* plant (250 mg/kg) decreased concentrations of malondialdehyde (MDA), secondary products generated by lipid peroxidation. Moreover, it increased the activity of antioxidant enzymes (SOD and CAT) in rats with liver damage [74].

## 8. *Mucuna pruriens* and Nitric Oxide

NO is a gaseous molecule that can diffuse freely through cell membranes. NO is synthesized by a group of enzymes known as nitric oxide synthases (NOS) from L-arginine, oxygen, and different cofactors depending on the type of cells where the synthesis is carried out [75,76]. Three members of the NOS family of enzymes have been identified in mammals: neuronal NOS (nNOS), endothelial NOS (eNOS), and inducible NOS (iNOS); they exhibit distinctive functional and structural characteristics [77]. NO produces direct reactions with the body systems and is involved in the pathophysiology of many neurological disorders. These include epilepsy, schizophrenia, drug addiction, anxiety, and major depression [78]. Moreover, it has been reported that there is an increase of NO in plasma concentration in depressed patients with suicidal tendencies [79].

**Table 3:** Antioxidant effects of treatment with *M. pruriens* in various pathologies. SOD: superoxide dismutase; GSH: reduced glutathione; CAT: catalase; GPx: glutathione peroxidase; GST: glutathione-S-transferase; GR: glutathione reductase; MPTP: 1-Methyl-4-phenyl-1,2,3,6-tetra hydropyridine; PQ: paraquat; STZ: streptozotocin; BCA: bilateral carotid artery.

Species	Inducing Agent/Model	Treatment with *Mucuna pruriens*	Effect	Reference
Swiss mice	MPTP/Parkinson’s disease	100 mg/kg, p.o. for 7 days	↓ Lipid peroxidation ↑ GSH activity	Yadav et al., 2014 [45]
Swiss mice	PQ/ Parkinson’s disease	100 mg/kg, p.o. for 9 weeks	↓ Lipid peroxidation	Yadav et al., 2017 [80]
Swiss mice	MPTP/ Parkinson’s disease	50 mg/kg, i.p. for 2 weeks	↓ Lipid peroxidation ↑ SOD and CAT activity	Srivastava et al., 2022 [81]
Wistar rats	Aging	200 mg/kg, p.o. for 60 days	↓ Lipid peroxidation ↑ SOD, CAT, GPx, GST, and GR activity	Suresh et al., 2010 [66]
Wistar rats	STZ/ Diabetes	200 mg/kg, p.o. for 60 days	↓ Lipid peroxidation ↑ SOD, CAT, GSH, GPx, GST, and GR activity	Suresh et al., 2013 [82]
Wistar rats	BCA occlusion/ Ischemia	100 and 200 mg/kg, p.o. for 10 days	↓ Lipid peroxidation ↑ SOD, CAT, and GSH activity	Nayak et al., 2017 [26]
Wistar rats	Haloperidol/Tardive dyskinesia	100 and 200 mg/kg, i.p. for 21 days	↓ Lipid peroxidation ↑ SOD, CAT, and GSH activity	Pathan et al., 2011 [83]
Sprague Dawely rats	Cisplatin/Nephrotoxicity	200 and 400 mg/kg, p.o. for 7 days	↓ Lipid peroxidation ↑ SOD, CAT, and GSH activity	Modi et al., 2013 [84]
Wistar rats	Traumatic brain injury	50 mg/kg i.p. per day for 5 days	↓ Lipid peroxidation ↑ GSH	Mata-Bermudez et al., 2024 [37]

In support of this, inhibition of NOS by the administration of N-omega-nitro-L-arginine methyl ester (L-NAME, 10 mg/kg), an inhibitor of both nNOS and eNOS [85], and the administration of aminoguanidine (20 μg/μL, iNOS inhibitor) can prevent the development of behavior associated with depression in chronically stressed rats [86].

In this same context, treatment with alcoholic and aqueous extracts of *M. pruriens* (100 mg/kg, p.o.) significantly reduced the concentration of nitrites and the expression of iNOS in the nigrostriatal region of mice with PD [80]. Extracts of *M. pruriens* (100 µg/mL) have reduced NO production in LPS-stimulated RAW 264.7 macrophages [87]. Likewise, a 400 μg/mL concentration of *M. pruriens* significantly inhibited NO production in BV2 microglial cells stimulated with lipopolysaccharide (LPS) [88]. The evidence suggests that reducing NO levels can induce antidepressant-like effects and can be used as a strategy to improve the clinical efficacy of serotonergic antidepressants [78].

## 9. *Mucuna pruriens* and Cortisol

One of the hypotheses about the generation of depressive disorders denotes the deregulation of the hypothalamic–pituitary–adrenal (HPA) axis. The HPA axis is important due to its control over cortisol, one of the main regulation systems of the body in stressful situations. Part of this hypothesis is based on the results of studies carried out on patients with depression whose plasma cortisol levels have been assessed, finding significant fluctuations concerning patients without depression [89].

Various studies have focused on investigating the role of the HPA axis in patients with depression. The outcomes outlined that the HPA axis is activated in stressful situations and stimulates the production of corticotropin-releasing hormone (CRH) by the hypothalamus. Thus, it stimulates the secretion of adrenocorticotropin (ACTH) from the pituitary gland, which induces glucocorticoid secretion from the adrenal gland’s cortex, specifically cortisol [90].

Cortisol is the best-known glucocorticoid; it is produced in the adrenal cortex by the action of the enzyme 11-β hydroxylase that converts 11-deoxycortisol into cortisol. It has a half-life of 90 min, and its concentration is 10–20 µg/dL in plasma. Cortisol is a lipophilic molecule that can cross the blood–brain barrier and activate specific receptors in the hippocampus and other regions [91]. Recent studies established that almost 80% of patients diagnosed with major depressive disorder have impaired cortisol secretion [92], ACTH secretion, and elevated levels of CRH in the cerebrospinal fluid [93]. Likewise, a considerable increase in corticosterone has been observed in animal models of depression [94].

Previous studies indicate that treatment for 20 days with *M. pruriens* reduced serum corticosterone concentrations in chronically stressed rats [27,95]. A clinical trial in infertile patients reported that treatment with *M. pruriens* seed powder (5 g) orally once daily for three months reduced serum cortisol concentrations in the seminal fluid [96]. Taken together, the data postulate that *M. pruriens* can reduce cortisol levels in different experimental models, which is pivotal evidence for the treatment of depression since hypercortisolemia is closely linked to the appearance of depressive episodes [97].

## 10. *Mucuna pruriens* and Inflammatory Response

Systemic inflammation and neuroinflammation are considered essential and crucial components of the pathogenesis of neurodegenerative and psychiatric disorders [98]. In patients with major depressive disorder, an increase in the release of proinflammatory cytokines such as interleukin-1 (IL-1) and tumor necrosis factor-alpha (TNF-α) [99], chemokines, and cell adhesion molecules, including α-1 acid glycoprotein, α-1 antichymotrypsin, haptoglobin, human macrophage chemoattractant protein-1 (MCP-1), soluble intercellular adhesion molecule-1 (sICAM-1), and E-selectin [100], has been observed. Previous reports revealed that the administration of LPS in rats increases the immobility time in the forced swim test and the tail suspension test; it also increases the expression of pro-IL-1β and IL-1β proteins and induces the activation of the NLRP3 inflammasome in the mouse hippocampus [98]. Likewise, in a model of depression induced by corticosterone, an increase in serum levels of IL-6 and IL-1β was found [101], indicating that inflammatory processes are involved in the development of depressive disorders.

Previous research has established that anti-inflammatory treatment can produce antidepressant effects [102]. Treatment with Baicalin, a flavonoid compound extracted and purified from the dried roots of *Scutellaria baicalensis*, increases preference for sucrose consumption and decreases immobility time in the forced swim test and the tail suspension test. These data were associated with a considerable decrease in levels of proinflammatory cytokines (IL-1β, IL-6, TNF-α) in serum and hippocampal homogenate of mice subjected to chronic stress [103]. Furthermore, it has been postulated that *M. pruriens* has numerous anti-inflammatory properties according to diverse animal models [20,104]. This was demonstrated by a study where *M. pruriens* (400 μg/mL) was administered, resulting in a decreased release of inflammatory mediators such as IL-1β, IL-6, and TNF-α in LPS-stimulated BV2 microglial cells [88]. A recent study revealed that extracts of *M. pruriens* (100 µg/mL) showed decreased expression of cyclooxygenase-2 (COX-2), JNK/p-JNK, and ERK1/2/p-ERK1/2 in RAW 264.7 macrophages stimulated with LPS [87]. Moreover, *M. pruriens* has reduced carrageenan-induced paw edema (acute inflammation model) [105]. In this same sense, Tavares et al. (2020) indicated that treatment with *M. pruriens* decreased behavior associated with depression in obese rats with a subsequent decrease in the expression of IL-6 in the hippocampus [24].

## 11. Clinical Trials of *Mucuna pruriens*

It is worth noting that there are very few reports of humans using *M. pruriens* (Table 4). However, as early as 1995, the Parkinson’s Disease Study Group reported significant reductions in MDS-UPDRS scores using an extract of *M. pruriens* in 60 patients with PD [106]. In 1997, Rani used a mixture of various medicinal plants, including *M. pruriens*, minerals, and high-energy carbohydrate molecules called Revivin, in 251 patients who needed a rejuvenating agent to enhance performance and focus and decrease exhaustion, stress, and sexual dysfunction during the convalescence of a chronic disease. Over four weeks, patients were given one capsule daily; all reported good relief in various symptoms (69–77%), and the medication was well tolerated [107]. Nagashayana (2000) [108] used a mixture of four medicinal plants, including *M. pruriens*, in patients with mild to moderate PD and found significant improvements in activities of daily living and motor symptoms. Subsequently, the use of *M. pruriens* extracts in the treatment of male infertility and PD was reported. Regarding the first aspect, Ahmad (2008) reported that *M. pruriens* seed powder (5 g/day) administered orally to infertile men aged 22 to 45 years for three months considerably reduced lipid peroxidation, increased spermatogenesis, improved sperm motility, and restored the levels of cholesterol, phospholipids, total lipids, triglycerides, and vitamins A, C, and E in seminal plasma [19]. Shukla reported that *M. pruriens* lowered the hormonal levels of FSH and PRL and increased the hormonal levels of T and LH and the catecholamines: adrenaline, norepinephrine, and dopamine [63,96]. Additionally, Gupta reported that *M. pruriens* enhanced the blood serum’s and seminal plasma’s altered alanine, citrate, glycerophosphocholine, histidine, and phenylalanine contents [109].

Regarding the second aspect, most *M. pruriens* clinical trials have involved individuals with Parkinson’s disease because of the plant’s high L-Dopa concentration. As a result, *M. pruriens* is suggested as an alternate treatment for this condition in several reviews that can be found in the literature [20,114,115,116]. Compared to a standard L-Dopa dose (200/50 mg, L-Dopa/CD), rapid-acting initial kinetics and a slightly longer duration of therapeutic response were observed in 8 patients receiving 15 or 30 g of *M. pruriens* seed powder. *M. pruriens* showed a rapid onset of action and longer duration of effect compared to standard L-Dopa [113]. Cilia (2017) conducted a clinical study to examine the safety and effectiveness of consuming a single-dose powder of *M. pruriens* made from toasted seeds without pharmacological processing. Compared to dispersible L-Dopa (3.5 mg/kg) combined with the dopa-decarboxylase inhibitor benserazide (DDCI), it was found that low doses of *M. pruriens* (12.5 mg/kg) exhibited a similar motor response, with fewer dyskinesias and adverse events (disruptions in blood pressure and heart rate and severity of dyskinesias); high doses (17.5 mg/kg) induced more significant motor improvement at 90 and 180 min, with a longer duration, fewer dyskinesias, and fewer adverse events [17].

Moreover, the tolerability profile of high doses was better, and the clinical effects were comparable to those of L-Dopa alone at the same dose. Subsequently, during a 16-week evaluation period, the same authors assessed 18 patients; daily consumption of *M. pruriens* resulted in gastrointestinal side effects and a progressive decline in motor performance in seven patients. Nonetheless, the clinical response was comparable to the L-Dopa/CD preparation (250/25 mg) for those who could tolerate *M. pruriens* powder [18].

Radder (2019) reported the case of a patient self-medicating with *M. pruriens*, who showed marked motor improvement after the administration of a DDCI [111], which coincides with the observations of Contin [112], who studied two patients with PD and reported that L-Dopa bioavailability was significantly lower after *M. pruriens* administration compared with standard L-Dopa formulations. Meanwhile, Sakata (2024) administered *M. pruriens* and a commercial formulation of L-Dopa/CD to seven patients and found higher plasma levels of L-Dopa and longer-lasting symptom relief after the administration of *M. pruriens* compared to the commercial formulation [110].

All of the above reinforces the need to conduct more clinical studies on the administration of *M. pruriens*.

## 12. Conclusions and Perspectives

*M. pruriens* extracts have been used for therapeutic purposes in animal models of diseases such as cancer, diabetes, skin infection, erectile dysfunction, anemia, and hypertension. Our group has documented that *M. pruriens* extract prevented depression-like behavior induced by mild traumatic brain injury in a rat model, which was associated with preventing lipid peroxidation in the midbrain and increasing levels of reduced GSH in the cerebral cortex.

Furthermore, the literature widely validates that *M. pruriens* extracts can be used in PD; multiple preclinical trials validate it. Nevertheless, there is little evidence in clinical trials. However, significant improvement has been found in clinical trials, although it is noted that the sample sizes have been small. It is also worth noting that several studies suggest that a DDCI should be administered together with *M. pruriens* extracts. Additionally, several clinical studies of *M. pruriens* for treating male infertility, in which the sample sizes are larger, report excellent results and do not point to outside effects.

In conclusion, the *M. pruriens* plant is a potential candidate for treating depressive disorders. This represents a safe and affordable therapy. Its bioactive components, such as genistein, ursolic acid, gallic acid, β-sitosterol, L-Dopa, and 5-TH, outline great antidepressant effectiveness in various experimental models. In addition, *M. pruriens* increases the production of norepinephrine and decreases cortisol, NO, ROS, and anti-inflammatory cytokines. Therefore, the adjacent mechanisms of action are worthy of further investigation in preclinical models. Moreover, increasing the number and sample sizes of clinical studies is necessary, especially in treating depression.

## Figures and Tables

**Table 4:** Clinical studies and case reports using M. pruriens extracts. PD: Parkinson’s Disease; CD: carbidopa; DDCI: dopa-decarboxylase inhibitor; MDS-UPDRS: Movement Disorder Society–Unified Parkinson’s Disease Rating Scale.

Studied Population	Main Results	Conclusions	Reference
7 patients with PD (5 women, 2 men); mean age 66.1 years, mean disease duration 11.7 years, mean L-Dopa treatment 10.4 years	Each patient received a single dose of 100/10 mg L-Dopa/CD tablets or 11 g of *M. pruriens*. Plasma L-Dopa and metabolites were measured along with the onset and duration of clinical effect. *M. pruriens* doubled the clinical effect duration compared to L-Dopa/CD. Plasma L-Dopa levels were significantly higher with *M. pruriens*, and no increase in dyskinesia was observed.	*M. pruriens* is an effective, low-cost alternative for PD treatment, offering faster, longer-lasting symptom relief compared to standard L-Dopa/CD without increasing dyskinesia.	Sakata et al., 2024 [110]
48-year-old woman with PD	For several years, her treatment consisted of *M. pruriens* 800 mg four times daily, containing approximately 160 mg L-Dopa/day. CD was added to *M. pruriens*, resulting in marked motor improvement (documented on video and using MDS-UPDRS motor scores).	Adding a dopa-decarboxylase inhibitor (DDCI) to *M. pruriens* could be suitable for patients reluctant to start L-Dopa.	Radder et al., 2019 [111]
14 patients with advanced PD	Patients with motor fluctuations and dyskinesias received *M. pruriens* (45–100 g/day) and L-Dopa/CD (500–1000 mg/day) in a randomized order and crossover design over a 16-week period. Daily *M. pruriens* intake showed variable tolerance and similar efficacy to commercial levodopa in some patients.	Tolerance remains challenging; further studies are needed to optimize dosing and formulation.	Cilia et al., 2018 [18]
18 patients with advanced PD	Patients’ treatments were L-Dopa/DDCI 3.5 mg/kg; *M. pruriens*-high-dose (17.5 mg/kg); *M. pruriens*-low-dose (12.5 mg/kg); L-Dopa without DDCI; and *M. pruriens*/DDCI. High doses of *M. pruriens* resulted in significant motor improvement and fewer adverse effects compared to L-Dopa.	The clinical effects of *M. pruriens*-high-dose were comparable to L-Dopa with a better tolerance profile.	Cilia et al., 2017 [17]
2 patients with PD	Patients received 100 mg/25 mg of L-Dopa/CD (patient 1) or benserazide (patient 2) versus 100 mg L-Dopa from *M. pruriens* in two different sessions. L-Dopa bioavailability was significantly lower after *M. pruriens* administration compared with standard L-Dopa formulations.	Reduced L-Dopa bioavailability in *M. pruriens* preparations is expected due to the absence of DDCI.	Contin et al., 2015 [112]
180 infertile patients and 50 controls	Patients were administered *M. pruriens* (5 g/day) orally in a single dose with milk for three months. Clinical variables in seminal plasma and blood serum improved post-therapy in infertile men.	*M. pruriens* improved fertility-related variables in infertile men.	Gupta et al., 2011 [109]
60 infertile men under psychological stress	Patients were administered *M. pruriens* (5 g/day) orally in a single dose with milk for three months. Improved sperm count and motility and increased antioxidant levels in the seminal plasma of patients administered with *M. pruriens* were observed.	*M. pruriens* reduces stress and improves semen quality in infertile men by enhancing the antioxidant defense system.	Shukla et al., 2010 [96]
75 infertile men and 75 controls	The infertile men were prescribed *M. pruriens* (5 g/day), orally, in a single dose with milk for 3 months. Testosterone levels increased, and semen quality improved in the infertile men.	*M. pruriens* enhances fertility by regulating steroidogenesis and semen quality.	Shukla et al., 2009 [63]
60 infertile men and 60 controls	The infertile men were prescribed *M. pruriens* (5 g/day), orally, in a single dose with milk for 3 months. *M. pruriens* significantly inhibited lipid peroxidation, elevated spermatogenesis, and improved sperm motility. It also restored lipid profiles and vitamins A, C, and E, and fructose in seminal plasma.	Treatment with *M. pruriens* increased sperm concentration and motility in infertile men.	Ahmad et al., 2008 [19]
8 patients with PD	Patients were challenged with single doses of L-Dopa/CD (200/50 mg) and 15 or 30 g of *M. pruriens* in randomized order at weekly intervals. *M. pruriens* showed a rapid onset of action and longer duration of effect compared to standard L-Dopa.	*M. pruriens* may offer advantages in long-term Parkinson’s management without significantly increasing dyskinesia.	Katzenschlager et al., 2004 [113]
18 patients with mild to moderate PD, approx. 60 years old	A total of 13 patients received cleansing therapy (oleation, sudation, purgation, enema, and errhines) followed by palliative therapy (*M. pruriens*, 4.5 g; *H. reticulatus*, 0.75 g; *W. somnifera*, 14.5 g; and *S. cordifolia*, 14.5 g; suspended in 200 mL of warm milk, twice daily an hour prior to any meals) for 56 days, and a group of 5 patients received only palliative therapy for 84 days. Patients who received both therapies showed significant improvements in activities of daily living and motor symptoms (tremors, bradykinesia, rigidity, and cramps) compared to those who received palliative therapy alone.	Cleansing therapy appeared to facilitate better absorption of Ayurvedic medications in the gastrointestinal tract, enhancing the effects of subsequent palliative therapy.	Nagashayana et al., 2000 [108]
251 patients (196 men, 55 women), mean age 44 years	Patients received one capsule of Revivin (a herbomineral capsule composed of *Withania sominfera*, *Asparagus racemosus*, *Glycyrrhiza glabra*, *M. pruriens*, *Myristica fragrans, Anauchus pyrethrum*, *Ipomoea digitata*, *Sida cordifolia*, zinc ash complex, and high-energy carbohydrate molecules) daily for 4 weeks. All patients treated with the drug reported good improvement in the various symptomatology of general weakness of appetite, sleep, mood, and concentration. The overall improvement ranged between 69 and 77%.	Revivin, containing *M. pruriens*, was effective and well tolerated for various symptoms.	Rani et al., 1997 [107]
60 patients with PD (46 men, 14 woman), mean age 59 years	HP-200 doses were initiated at 7.5 g thrice daily and increased at the week 2 and week 4 visits to obtain the optimal response. HP-200 (a powder of *M. pruriens*, L-Dopa-33.33 mg/g) showed significant reductions in MDS-UPDRS scores over 12 weeks without adverse effects.	HP-200, derived from *M. pruriens* in Ayurvedic medicine, was effective for PD symptom management.	HP-200 in Parkinson’s Disease Study Group, 1995 [106]

## Data Availability

Not applicable.
